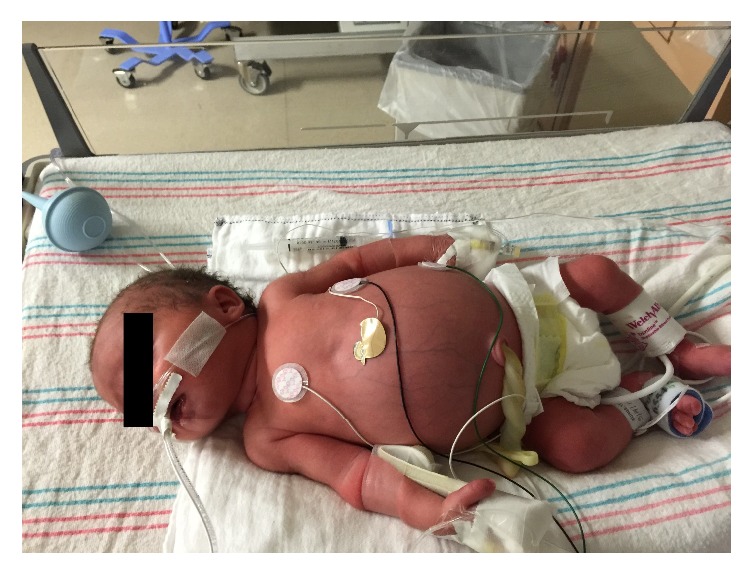# A Novel Mutation in ACTG2 Gene in Mother with Chronic Intestinal Pseudoobstruction and Fetus with Megacystis Microcolon Intestinal Hypoperistalsis Syndrome

**DOI:** 10.1155/2017/9146507

**Published:** 2017-12-14

**Authors:** Julie R. Whittington, Aaron T. Poole, Eryn H. Dutta, Mary B. Munn

**Affiliations:** ^1^Department of Obstetrics and Gynecology, University of Arkansas for Medical Sciences, Little Rock, AR, USA; ^2^Department of Obstetrics and Gynecology, Naval Medical Center Portsmouth, Portsmouth, VA, USA; ^3^Department of Obstetrics and Gynecology, Naval Medical Center Camp Lejeune, Camp Lejeune, NC, USA; ^4^Department of Obstetrics and Gynecology, University of Texas Medical Branch, Galveston, TX, USA

## Abstract

*Background. *A novel mutation in the ACTG2 gene is described in a pregnant patient followed up for chronic intestinal pseudoobstruction (CIPO) during pregnancy and her fetus with megacystis microcolon intestinal hypoperistalsis syndrome (MMIHS).* Case. *24-year-old gravida 1 para 1 with CIPO and persistent nausea and vomiting in pregnancy, admitted at 28 weeks of gestation. Ultrasound revealed a fetus measuring greater than the 95th percentile, polyhydramnios, and megacystis. At delivery, the newborn was noted to have an enlarged bladder, microcolon, and intolerance of oral intake. Genetic testing of mother and child revealed a novel mutation in the ACTG2 gene (C632F>A, p.R211Q).* Conclusion. *This is the first case in the literature describing a novel mutation in ACTG2 associated with visceral myopathy affecting both mother and fetus/neonate. Visceral myopathy should be included in the differential diagnosis of megacystis diagnosed by ultrasound, and suspicion should increase with family history of CIPO or MMIHS.

## 1. Introduction

Familial visceral myopathy is a rare condition characterized by smooth muscle cell dysfunction causing intestinal and genitourinary disorders. As of 2015, only 47 cases of visceral myopathy due to confirmed ACTG2 (2p13.1) gene mutations have been described [1]. Multiple phenotypes of ACTG2 related disorders have been described including megacystis microcolon intestinal hypoperistalsis syndrome (MMIHS), prune belly sequence, and chronic intestinal pseudoobstruction (CIPO). ACTG2 mutations are inherited in an autosomal dominant manner [1]. There can be variable involvement of bladder and intestine, and while penetrance of ACTG2 related disorders is complete, its expressivity can vary. MMIHS involves both functional intestinal obstruction which typically requires extensive surgical intervention for survival and prenatal diagnosis of an enlarged bladder [2]. ACTG2 encodes enteric smooth muscle actin, and affected individuals have been shown to have abnormal smooth muscle actin [3]. We describe a mother with CIPO and her fetus/neonate with prenatally diagnosed megacystis who both have a novel ACTG2 gene mutation.

## 2. Case

A 24-year-old gravida 2 para 1 presented at 28 weeks of gestational age with nausea, vomiting, and inability to tolerate oral intake. Her past medical history was significant for chronic constipation and need for intermittent self-catheterization as a child. Her prior pregnancy was complicated by a primary cesarean with postoperative ileus and clostridium difficile infection requiring bowel resection. She had a total of 5 prior surgeries on her intestine and colon throughout her life. Repeated laparotomy for abdominal pain is common to CIPO [4]. Her family history was significant for a brother with megacystis who had bladder surgery as a child and required catheterization as a toddler. Her prior child was alive and well with no medical issues.

**Figure 1:**
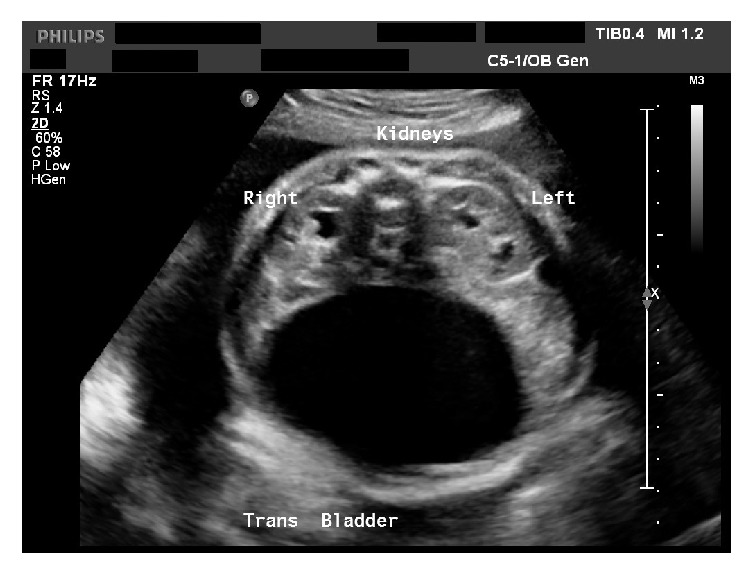


She was hospitalized for a total of 5 weeks during her second pregnancy beginning at 28-week gestational age and required nasojejunal feeds for nutrition. General surgery was consulted and computed tomography demonstrated ileus pattern with no obvious evidence of obstruction. While hospitalized, an ultrasound at 31 weeks of gestation revealed a fetus measuring greater than the 95th percentile, polyhydramnios, and severe megacystis (Figure 1). She eventually delivered at 35 weeks of gestational age due to persistent intolerance of oral intake. Repeat cesarean delivery was performed.

At birth, her infant was noted to have an enlarged bladder, microcolon, and poor tolerance of oral intake (Figure 2). He required catheterization to drain his bladder. A colonic biopsy was performed which revealed ganglion cells were present, ruling out Hirschsprung's disease. He eventually required partial colonic resection with colostomy. He had a second abdominal surgery in his first 12 months of life with additional resection of bowel. Since this last abdominal surgery, he has had improved weight gain and tolerance of oral intake.

Genetic testing was performed on the mother and the infant, and they were both confirmed to have a novel heterozygous mutation in the ACTG2 gene (C632G>A, p.R211Q) on chromosome 2p13.1.

## 3. Discussion

Visceral myopathy is a rare condition that has recently been attributed to mutations in the ACTG2 gene. Poor prognosis has been associated with this condition, especially MMIHS. Urinary tract infection and bowel obstruction along with poor nutrition all contribute to the poor prognosis [5]. Early diagnosis and a multidisciplinary approach are important for the optimization of care. In a systematic review by Tuzovic et al., the ultrasonographic findings associated with MMIHS were reviewed. The most common findings were fetal megacystis (presenting finding in 88%), hydroureteronephrosis (68%), and normal to increased amniotic fluid volumes. [6]

We describe a novel mutation in the ACTG2 gene. Based on the maternal and neonatal presentations, this gene mutation may be associated with a milder phenotype than other patients with mutations in ACTG2. Wangler and Beaudet describe 5 probands that were heterozygous for ACTG2 variants. It was noted that the familial cases had a milder disease than de novo cases [1]. Visceral myopathies need to be considered in the setting of megacystis, especially with accompanying gastrointestinal abnormalities or history of affected family members. As the inheritance pattern can be autosomal dominant, a complete family history of the fetus or neonate and their family is indicated in cases of suspected MMIHS.

## Figures and Tables

**Figure 2:**